# Use and acceptance of complementary and alternative medicine among medical students: a cross sectional study from Palestine

**DOI:** 10.1186/s12906-019-2492-x

**Published:** 2019-04-02

**Authors:** Ahmad M. Samara, Ethar R. Barabra, Hala N. Quzaih, Sa’ed H. Zyoud

**Affiliations:** 1 Department of Medicine, College of Medicine and Health Sciences, An-Najah National University, Nablus, 44839 Palestine; 2 Poison Control and Drug Information Center (PCDIC), College of Medicine and Health Sciences, An-Najah National University, Nablus, 44839 Palestine; 3 Department of Clinical and Community Pharmacy, College of Medicine and Health Sciences, An-Najah National University, Nablus, 44839 Palestine

**Keywords:** CAM, Medical students, Knowledge, Attitude, Beliefs, Palestine

## Abstract

**Background:**

Teaching Complementary and alternative medicine (CAM) in medical schools is becoming prevalent worldwide. Few studies have been conducted to evaluate medical students’ knowledge and attitude toward CAM. Therefore, this study was designed to assess CAM knowledge, attitudes, and beliefs among Palestinian medical students.

**Methods:**

This study was developed in a cross-sectional design. It targeted medical students at An-Najah National University, between January and April of 2018. We gathered the data from students using a questionnaire printed as a hard copy. Medical students of both sexes in their 4th, 5th, or 6th year of studies were included in the survey. The questionnaire consisted of 3 sections: demographic characteristics and detailed practices of the participants, their attitude and held beliefs towards CAM, and their knowledge on CAM. Mann-Whitney U Test and Kruskal-Wallis Test were used to test if there were differences between knowledge about CAM and the characteristics of the participants.

**Results:**

Of the 300 medical students who were offered the questionnaire, 251 students (43.8% male and 56.2% female) were included in the final analysis. Out of a maximum of 8 points, the mean knowledge score of the participants was 2.0 ± 1.6. The Kruskal-Wallis test showed a statistically significant difference in overall knowledge score among students of different year groups, with students at lower levels obtaining higher scores (*P* < 0.001). Additionally, the highest knowledge scores were found in students with low income, and students who came from the Palestinian refugee camps (*P* < 0.001). Students frequently recommended CAM modalities, with herbal medicine being the most recognized and used CAM modality and Ayurvedic medicine being the least recognized and recommended one. Social media was the most popular source of information about CAM, cited by 72.9% of the participants. Participants generally had a good attitude towards CAM but held varying beliefs about it.

**Conclusions:**

In the current study, a knowledge gap regarding CAM was found among medical students in our sample, despite their good attitude towards the subject. Also, there was a general acceptance to include materials on CAM within the curriculum of medical students.

**Electronic supplementary material:**

The online version of this article (10.1186/s12906-019-2492-x) contains supplementary material, which is available to authorized users.

## Background

Complementary and Alternative Medicine (CAM) is gaining more importance around the world, both as a topic for research and a used modality. This trend is also true in developed countries. CAM, as a general term, encompasses various practices, products, and medical or health systems that are not commonly recognized in the same way conventional medicine is [1]. In a literal sense, the term “alternative” refers to any practice that replaces medical therapy, and the term “complementary” refers to any practice that completes the medical therapy [2, 3]. However, it is important to note that CAM, as a general term, can include all sorts of practices and modalities that are used in agreement with its literal description but can vary widely in their effectiveness, approval for medical use, and the evidence of their benefit or harm. Therefore, a positive attitude towards CAM, as hinted on in this study, does not always translate into encouragement of CAM use and popularization [4, 5]. Instead, it indicates the evidence-based understanding of CAM and the awareness of its common uses and misuses, which can help incorporate its modalities in the community, in a healthy way [6, 7].

CAM involves five categories of practices [3, 8]. The first category involves the use of natural products, such as herbs, probiotics, antioxidants, and chondroitin sulfate. The natural product that is used the most for CAM is fish oil, which is a substance rich in omega 3 and is thought to have some protection against several problems like heart diseases. The second category involves mind-body therapy, which uses techniques that enhance the ability of the mind to influence the function of the body and improve health. Examples of this category include hypnotherapy and yoga. The third category is Alternative Medical Systems, examples of which include homeopathy and naturopathic medicine. It also includes using traditional medical systems from different countries like Ayurved, which is a form of CAM used in India, and Traditional Chinese Medicine (TCM) which includes acupressure and acupuncture. The fourth category is manipulation and body-based methods which are based on the manipulation and movement of one or more of the body parts as seen in body massage and sport. The fifth category is Energy Therapy, which is based on the idea that energy fields surround and penetrate the human body. Some people, however, use types of CAM other than these five categories, such as religious rituals including prayer and reciting Quran.

CAM is popular among the general public [9, 10] but is less so among medical students [11], despite that it is reasonable for medical students, as physicians in the making, to have adequate knowledge of and good attitude towards CAM. The two most commonly used CAM modalities on a global scale, including university students, are biologically based modalities (which include herbals) and mind-body medicine modalities (which include yoga, spiritualities, meditation, and hypnosis) [12–21]. There have been a rising number of studies investigating medical students’ knowledge and attitude regarding CAM in recent years. For example, two recent studies from Saudi Arabia that assessed knowledge and attitude regarding CAM among medical students [22, 23] concluded that there was a knowledge gap regarding CAM among students, despite their positive attitude towards the topic. Another study conducted at two universities in the United States investigated the views of medical students and students in other health-care professions on CAM [24] and reported that medical students received the least amount of education about CAM and doubted the usefulness of CAM the most among all health professions students.

In Palestine, many studies on CAM were conducted, targeting different kinds of populations which include hypertensive patients [25], diabetes mellitus patients [26], hemodialysis patients [27], cancer patients [28], and the general public [29]. CAM use was found to be common in Palestine; in one study, 67% of men and 77% of women documented using CAM within the past year to treat their illnesses [29]. However, the data on CAM use among Palestinian medical students is still lacking. Additionally, many studies were conducted to investigate herbal therapies use among various populations such as cancer patients [30], geriatric population [31], and pregnant women [32, 33], but none targeted university students.

In this study, we aimed to assess medical students’ knowledge, perceptions, and interest in CAM in Palestine, where many practices and modalities of CAM are considered vital components of both culture and religion [34–39]. Also, we aimed to evaluate the association between students’ characteristics and their knowledge on CAM. Having knowledge about CAM can positively affect the attitude towards it, which, in turn, can facilitate including this subject within the curriculum taught in medical schools. Also, determining medical students’ knowledge, perception and attitude regarding CAM can help incorporate these systems in the health care-system at large, in a healthy manner, for today’s students are the doctors of the next generation and will influence creating and applying related policies in the future. In light of this, the results of the current study provide important information regarding CAM situation that can help improve this situation. They can also function as a necessary reference point for implementing the appropriate policies and recommendations and assessing their effectiveness with further studies. Improvement of this field, which is closely related, if not an integral part of the health-care system, can have a positive effect on medical education, as well as patients’ care, by addressing the misconceptions and knowledge gap regarding CAM and the use and usefulness of its modalities and establishing the scientific bases for accepting and rejecting these modalities.

## Methods

### Study design

This is a cross-sectional study with data collected using a questionnaire.

### Population and setting

This study targeted medical students in their 4th, 5th or 6th years at An-Najah National University (ANNU) in Nablus in the West Bank, Palestine, which is one of the only two universities in the West Bank that have an established medical school in them. ANNU is the largest university in Palestine with approximately 22,000 full-time students. The university offers medical and non-medical education through its 11 different colleges [40]. Medical students at ANNU currently do not have any courses on CAM in their curriculum, though pharmacy students and students of other health sciences do have an elective course on CAM in their curricula. Therefore, medical students at ANNU have no proper exposure to the topic of CAM during their study years. The targeted medical years in this survey (4th, 5th or 6th years) are the years during which medical students receive their clinical training and start interacting with patients, which is also when they start getting all sorts of medical questions, including those related to CAM, which is why we decided to focus on this group of students in our study. We used a structured questionnaire that was designed to gather information about participant’s demographic data, attitude, beliefs, knowledge, and use of CAM.

### Sample size determination

There are approximately 500 medical students in the 4th, 5th or 6th years at ANNU, which is the number that were used to estimate the required sample size, using Raosoft sample size calculator: (http://www.raosoft.com/samplesize.html) with a confidence level of 95%, and a pre-determined margin of error of 5%. We assumed that the response distribution for each question would be 50% because we are not sure what to expect the results for each question to be. By using 50% as response distribution which gave a larger sample size for this research. The calculated sample size was 218, but we decided to survey 300 students in an attempt to insure a higher reliability. In this study, participants were selected by convenience sampling. Convenience sampling is a non-probability sampling technique where the target population that achieved certain practical criteria, such as availability at a given time, or the willingness to participation, easy accessibility, and geographical proximity to the researchers [41].

### Inclusion and exclusion criteria

Students were required to meet certain inclusion criteria that include carrying a Palestinian nationality, being a medical student at ANNU at least in the 4th years, choosing willingly to take part in the study. Those whose questionnaires were incompletely filled were excluded from the study.

### Data collection form

The questionnaire used in this study was designed after reviewing previous similar studies in the literature [15, 16, 21, 42–49] and was arranged in three sections. An additional file was supplied to illustrate comprehensive description of the study both in English and Arabic (Additional file 1). The first section contained questions on demographic characteristics such as age, gender, type of residence, level of education. The second section assessed the participants’ knowledge about CAM and the sources from which they acquired this knowledge. It contained eight statements testing uses, contraindications, and adverse effects of CAM. On this section, participants were asked to answer by choosing (true), (false), or (I do not know) for each statement. A knowledge score was generated by allocating one point for each correct response and zero points to incorrect or (I don’t know) answers. This score ranged from 0 to 8 with a higher score meaning better knowledge. Other questions in this section aimed to identify the sources used by students to get their knowledge on CAM. The third section investigated student’s attitudes toward CAM. The participants were asked if they personally used CAM, for what reason they used it, what factors affected whether they would recommend CAM or not, how satisfied they were with using CAM, and whether or not they suggested using CAM to relatives before. Also, participants were asked seven questions addressing the beliefs they held regarding CAM, particularly its safety, benefits, undesired or placebo effects and their confidence in its use. The participating medical students were required to answer on a Likert scale of 5, wherein 1 meant they strongly disagree and 5, they strongly agree. Moreover, this section included one question on obstacles limiting the proper implementation of CAM and the information that students should know regarding CAM like its uses, doses, side effects, drug interactions, effects on pregnancy. The language the questionnaire was presented to the participants in was Arabic. Three expert researchers in social studies assessed the survey instrument for face and content validity to assess the organization, completeness, appropriateness and logical sequence of the statements. Focused on their opinions, the questionnaire was modified and piloted on 25 medical students’ prior to its distribution among the study participants to assess the clarity and readability of the questionnaire.

### Data collection procedure

Data were collected using a self-administered paper-based questionnaire (see Additional file 1). Two trained medical students of the College of Medicine and Health Sciences at An-Najah National University administered the questionnaires to students attending clinical practicum before or after the clinical rotation. All participant students provided verbal informed consent prior to answering any question by reading the consent form, which was attached to the paper-based questionnaire. They were each allowed at least 15 min to complete the questionnaire.

### Ethical approval

Before initiating the study, we obtained the approval of the Institutional Review Board (IRB) at ANNU for this study. Participants were assured that participation in the study was voluntary. Security and privacy of responses, and confidentiality of the information were maintained. Additionally, the participants provided verbal informed consent to participate in the study, which is at the same level as written consent for cross-sectional studies like this in Palestine, as considered by the IRB.

### Statistical analysis

The Statistical Package for Social Sciences program version 18 (SPSS) was used for entering and analyzing the data. Data were expressed as frequencies and percentage for categorical variables and as means, medians, standard deviations (SD), and inter-quartile ranges for continuous variables. Mann–Whitney U and Kruskal-Wallis tests were implemented in the comparison of knowledge score differences between the participants’ categories on the bases of their characteristics. We assumed the level of statistical significance at *p* < 0.05.

## Results

### Demographic information

Of 300 questionnaires distributed among medical students of ANNU, 255 were filled and returned, giving a response rate of 85%. There was no significant difference between responding students and students how did not fill the questionnaire in terms of their socio-demographic characteristics. However, the final analysis was done on 251 questionnaires as 4 questionnaires were discarded due to incomplete information. The mean age of the participants in years was 22.5 with standard deviation of 1.14, and slightly more than half of the participants (56.2%) were females. The percentages of participants from 4th, 5th, and 6th study year were 39.0, 24.7, and 36.3%, respectively. The demographic characteristics of the participants are detailed in Table 1.

**Table 1:** Demographic characteristic of the participants

Variable	Number (%)
Age in years, mean (standard deviation)	22.5 (1.0)
Less than 23	109 (43.4)
23 or more	142 (56.6)
Gender	
Male	110 (43.8)
Female	141 (56.2)
Study year	
Fourth year	98 (39.0)
Fifth year	62 (24.7)
Sixth year	91 (36.3)
Residency	
City	145 (57.8)
Village	99 (39.4)
Palestinian refugee camps	7 (2.8)
Monthly income^a^	
Less than 500 JD	28 (11.2)
500–1000 JD	62 (24.7)
1000–2000 JD	70 (27.9)
More than 2000 JD	91 (36.3)

^a^1 Jordanian Dinar (JD) equals 1.41 US Dollar

### Knowledge of CAM

The results of CAM knowledge are summarized in Table 2. The mean knowledge score of the participants out of a maximum 8 points was 2.0 with a standard deviation of 1.6. The two most frequently correctly answered questions were about the safety of Spinach eating in patients with kidney disease (45.8% correct response rate) and the beneficial effect of Ginger for patients with premenstrual syndrome (34.3% correct response rate). The lowest correct response rates, on the other hand, were for questions about Echinacea’s effect on immunity (14.7%) and bran’s effect on the metabolism of digoxin (9.2%).

**Table 2:** Numbers and percentages of participants who answered correctly on the 8 knowledge questions

No.	Question^a, b^	Number (%)
1	Senna is contraindicated in the case of pregnancy and in children under twelve years old (T)	47 (18.7)
2	Spinach eating is safe for kidney patients (F)	115 (45.8)
3	Fenugreek rises the risk of increased blood sugar so it should be avoided in diabetics (F)	61 (24.3)
4	Garlic increases the risk of bleeding if used with warfarin (T)	73 (29.1)
5	Bronchoconstriction is the side effects of caffeine (F)	65 (25.9)
6	Ginger has effect in relieving premenstrual syndrome (T)	86 (34.3)
7	Echinacea is used for suppressing the immunity (F)	37 (14.7)
8	Digoxin use with bran increase the concentration of digoxin (F)	23 (9.2)

^a^Questions were adapted from Shraim et al. [47]

^b^correct answers set alongside each statement; T stands for true and F stands for false

Also, Table 3 shows the relationship between medical students’ characteristics and their knowledge score. There was no association between the participants’ age or gender and their knowledge score as evident by the *p* values for these two variables (both > 0.05). The Kruskal-Wallis test showed a statistically significant difference in overall knowledge score among students of different year groups, with students at lower levels (i.e. fourth- and fifth-year students) obtaining higher scores (*p*-value< 0.001). Additionally, The highest knowledge scores were found in students with low income, and students who came from the Palestinian refugee camps (*p*-value< 0.001).

**Table 3:** Relationship between characteristics of participants and their knowledge scores

Variable	Median [Q1-Q3]	*P* value^#^
Age in years		
Less than 23	2 [1–4]	0.453^a^
23 or more	2 [1–3]	
Gender		
Male	2 [1–3]	0.336 ^a^
Female	2 [1–3]	
Study year		
Fourth year	2 [1–4]	**<0.001** ^b^
Fifth year	2 [1–4]	
Sixth year	1 [0–2]	
Residency		
City	1 [0–3]	**<0.001** ^b^
Village	2 [1–4]	
Palestinian refugee camps	3 [0–4]	
Monthly income^c^		
Less than 500 JD	3.5 [2–5]	**<0.001** ^b^
500–1000 JD	2 [0–3]	
1000–2000 JD	2 [1–3]	
More than 2000 JD	1 [0–3]	

^a^*p*-value is calculated using Mann-Whitney U test

^b^*p*-value is calculated using Kruskal-Wallis test

^c^1 Jordanian Dinar (JD) equals 1.41 US Dollar

^#^Bold text indicates a statistically significant difference with a p-value less than 0.05

### Types of CAM

The participants’ familiarity with, use of, and attitude towards 17 types of CAM were assessed. The results of this assessment are summarized in Table 4. Herbal medicine and hypnosis were the two most familiar CAM types and were recognized by 79.3 and 78.5% of the participants, respectively, whereas Ayurvedic medicine and biofeedback were the two least recognized CAM types, by 15.9 and 19.5%, respectively. As for personal use, herbal medicine and massage therapy came at the top with 46.2% of the participants reported previously using each of these two types of CAM, whereas homeotherapy and Ayurvedic medicine came last as they were previously used only by 4.8 and 6.4% of the participants, respectively. When asked if they would advise patients to use different types of CAM, participants showed a positive attitude towards massage therapy and herbal medicine more frequently than any other CAM type, and they were least likely to advise using Ayurvedic medicine and traditional African healing.

**Table 4:** Types of complementary and alternative medicine (CAM) and participants’ knowledge and attitude regarding them

Types of CAM	Is it familiar to you? N (%)	Have you ever used it? N (%)	Would you advise using it to patients? N (%)
Homeopathy	63 (25.1)	12 (4.8)	49 (19.5)
Naturopathy	156 (62.2)	81 (32.3)	92 (36.7)
Acupuncture	195 (77.7)	46 (18.3)	56 (22.3)
Ayurvedic medicine	40 (15.9)	16 (6.4)	22 (8.8)
Aromatherapy	70 (27.9)	37 (14.7)	32 (12.7)
Chiropractic	163 (64.9)	41 (16.3)	102 (40.6)
Faith healing	151 (60.2)	75 (29.9)	71 (28.3)
Massage therapy	195 (77.7)	116 (46.2)	136 (54.2)
Traditional African healing	58 (23.1)	26 (10.4)	26 (10.4)
Iridology	100 (39.8)	50 (19.9)	42 (16.7)
Hypnosis	197 (78.5)	64 (25.5)	75 (29.9)
Meditation	173 (68.9)	68 (27.1)	100 (39.8)
Yoga	171 (68.1)	72 (28.7)	104 (41.4)
Reflexology	65 (25.9)	34 (13.5)	28 (11.2)
Energy Medicine	97 (38.6)	49 (19.5)	63 (25.1)
Herbal medicine	199 (79.3)	116 (46.2)	123 (49.0)
Biofeedback	49 (19.5)	29 (11.6)	29 (11.6)

When asked about CAM modalities they would recommend without being limited to the types mentioned before (Table 5), exercise came at the top recommended by 84.5% of the participants, followed by massage (79.3%), honey (71.7%), and nutritional complements (70.5%). Reflexology (13.5%), aromatherapy (14.3%), bloodletting (16.3%), and animal product (17.5%), on the other hand, were the least likely modalities to be recommended.

**Table 5:** Types of Complementary and alternative medicine (CAM) and the number distribution of participants who recommend them

Item	Number (%)
Exercise	212 (84.5)
Complements	177 (70.5)
Honey	180 (71.7)
Message	199 (79.3)
Herbs	148 (59.0)
Quran	163 (64.9)
Fasting	163 (64.9)
Praying	161 (64.1)
Hejama	96 (38.2)
Zamzam water	122 (48.6)
Remedies	133 (53.0)
Music	116 (46.2)
Chiropractic	96 (38.2)
Acupuncture	81 (32.3)
Aromatherapy	36 (14.3)
Bloodletting	41 (16.3)
Cauterization	60 (23.9)
Animal products	44 (17.5)
Reflexology	34 (13.5)

### Sources of CAM knowledge

Table 6 presents knowledge sources used by students to know about CAM. Social media was the most popular source for CAM knowledge among the participating students, reported by 72.9% of the participants as a primary source for such knowledge, followed by the internet (66.5%) and relatives (54.2%). Whereas only a third of the participant acquired their knowledge on CAM from a doctor or the university, and even less than that acquired this knowledge from scientific magazines (29.1%) or books (16.3%).

**Table 6:** Information sources used by participants as their source of knowledge on complementary and alternative medicine (CAM)

Source	Number (%)
Doctor	83 (33.1)
Relatives	136 (54.2)
Pharmacist	75 (29.9)
Social media	183 (72.9)
TV	114 (45.4)
Mosque	39 (15.5)
Friends	94 (37.5)
Internet	167 (66.5)
Scientific magazines	73 (29.1)
Religious books	38 (15.1)
School	63 (25.1)
University	84 (33.5)
Scientific books	41 (16.3)
Others	22 (8.8)

### Attitude towards CAM

Table 7 presents the percentages of participants who answered positively to ten questions related to their attitude toward CAM. All ten questions received positive responses by more than half of the participants reflecting a generally good attitude towards CAM among the participating students. For example, 72.5% agreed that patients have the right to choose between CAM and orthodox medicine and 68.5% agreed that it is necessary to ask all patients about any previous CAM use. Also, more than two-thirds of the participants believed than CAM is beneficial to the health-care system.

**Table 7:** Participants’ attitude towards complementary and alternative medicine (CAM)

Question^a^	Number (%)
Do you believe that CAM is beneficial to healthcare?	170 (67.7)
As a future doctor, will you recommend CAM to a patient?	146 (58.2)
Do you agree that patients have right to choose between CAM and orthodox medicine?	182 (72.5)
As a future doctor, will you encourage the use of CAM together with orthodox medicine?	140 (55.8)
Do you agree that CAM should be introduced in medical course?	160 (63.7)
Will you be ready to be trained more on CAM after becoming a doctor?	131 (52.2)
Do you agree that it’s necessary to ask every patient of previous usage of CAM during history taking?	172 (68.5)
As a future doctor, will you ask patient of previous CAM use?	168 (66.9)
Do you agree that It is necessary for a doctor to have good knowledge of CAM?	166 (66.1)
As a future doctor, will you have a positive reaction should a patient ask you to recommend a CAM?	146 (58.2)

^a^Questions were adapted from Ameade et al. [49]

### Beliefs about CAM

The results of participants’ opinion on 7 statements related to beliefs about CAM are presented in Table 8. The most agreed upon statement among participants was one that emphasized the need for scientific evaluation before using CAM with 72.5% of the participants answering between “agree” and “strongly agree”, followed by a statement highlighting the importance of asking the patient about any CAM modality use (62.9% between “agree” and “strongly agree”). A statement claiming that CAM result were due to a placebo effect was the most controversial statement with 35.5% of the participants agreeing or strongly agreeing with the statement and 35.8% disagreeing or strongly disagreeing with it.

**Table 8:** Participant’s beliefs about complementary and alternative medicine (CAM)

Item	Agree	Strongly agree	Disagree	Strongly disagree	No opinion
All types of CAM are safe and have very few side effects	110 (43.8)	31 (12.4)	45 (17.9)	6 (2.4)	59 (23.5)
Conventional medicine doesn’t offer the patient benefits offered by alternative medicine	50 (19.9)	22 (8.8)	82 (32.7)	34 (13.5)	63 (25.1)
Results of complementary medicine is mainly due to placebo effect	64 (25.5)	25 (10.0)	45 (17.9)	45 (17.9)	72 (28.7)
I have full trust to debate with patients about terms of alternative and complementary medicine	78 (31.1)	16 (6.4)	38 (15.1)	53 (21.1)	66 (26.3)
Complementary and alternative medicine not only cure the disease but will improve general health in other hand	119 (47.4)	19 (7.6)	32 (12.7)	18 (7.2)	63 (25.1)
The doctor should continuously question whether the patient was used modalities of alternative medicine	115 (45.8)	43 (17.1)	35 (13.9)	21 (8.4)	37 (14.7)
We need scientific evaluation before use complementary and alternative medicine	117 (46.6)	65 (25.9)	17 (6.8)	11 (4.4)	41 (16.3)

### Motivating and limiting factor regarding CAM recommendation

Table 9 presents the percentages of participants who considered certain factors to play a motivational role for them to recommend CAM use. The scientifically proven efficacy of a CAM product was the most cited factor with 80.5% of the participants citing it, followed by the CAM modality having a limited number of side effects (78.5%) and the positive feedback on the modality by the patients (72.1%).

**Table 9:** Motivating factors reported by the participants toward recommendations for complementary and alternative medicine use

Item	Number (%)
Product efficacy is scientifically proven	202 (80.5)
Positive responses from patients on the effectiveness of the product	181 (72.1)
Fewer side effects	197 (78.5)
Less expensive (Cheaper)	157 (62.5)
Publicity of the product	104 (41.4)
Highest profit	56 (22.3)
Incentives from manufacturers	73 (29.1)
Others	35 (13.9)

As for barriers that limit the use of CAM as perceived by the participants (Table 10), the lack of scientific evidence in regard to the use of CAM was the most cited factor, recognized by 76.6% of the participants, followed by the lack of evidence on the safety of CAM (by 72.9%) and the lack of scientific knowledge on CAM (by 72.1%).

**Table 10:** The barriers that limit the appropriate use of complementary and alternative medicine as reported by the participants

Item	Number (%)
The members that trained to use CAM are small number	163 (64.9)
Lack of scientific knowledge in CAM	181 (72.1)
Deficiency of scientific evidence to use CAM	192 (76.5)
Lack of safe sources of data	183 (72.9)
Need a long time of therapy	80 (31.9)
There is no time	91 (36.3)
Lack of interest in CAM	154 (61.4)
There is no hurdle	58 (23.1)

## Discussion

The strategic location of Palestine at the cross-point of Asia, Africa and Europe is a crucial determining factor for its wide range of CAM modalities. This fact puts additional pressure on community physicians since they are generally recognized and trusted as a source of scientific information in the eyes of their patients. Therefore, we set out to investigate the knowledge, attitude, beliefs, and perception of CAM among medical students, for they are the future holders of this responsibility. As far as we know, this was the first study to target medical student regarding their stand on CAM in Palestine.

We found that medical students’ knowledge on CAM was lacking with a mean knowledge score of 2.0 ± 1.6 (out of a maximum 8 points). Also, students frequently recommended CAM modalities, with herbal medicine being the most familiar and used CAM modality and Ayurvedic medicine being the least familiar and recommended one. Social media was the most popular source of information about CAM, cited by 72.9% of the participants and participants reported a good attitude towards CAM but held varying beliefs about it.

The existing literature demonstrates a difference between medical students from different countries in term of their knowledge on CAM. In a local study done in Saudi Arabia, magnetic therapy and spiritual healing were the most known CAM modalities, whereas the most known CAM modalities in Turkey were herbal medicine and acupuncture [4]. In Kuwait, prayer/ Quran reciting and herbal medicine were the most popular types [5]. In our study, herbal medicine was the most common modality among medical students. This could be due to the popularity of this modality in the culture of the area and among the general population. Ayurvedic medicine was the least common CAM type which, in turn, could be due to the fact that it is uncommon in the area in general.

Religious belief is a major element of the culture of the Palestinian society. Many herbal therapies are mentioned in the holy book (Quran) [50–54], such as garlic – which is thought to be beneficial as an antispasmodic, anti-allergic, diuretic, and digestive stimulant – and Ginger – which is generally used and thought to be helpful for anorexia, headache, abdominal pain originating from the bowel, constipation, diarrhea, and stomach disorder [55]. Another plants that are mentioned in the holy book (Quran) include onion (sūrat l-baqarah [2:61]), camphor (sūrat l-insān [76:5]), olive, fig (sūrat l-tīn [95:1]), squash (sūrat l-ṣāāt [37:146]), sweet basil (sūrat l-raḥmān [55:12]), grape (sūrat l-naḥl [16:11]), pomegranate (sūrat l-raḥmān [55:68]), among many others [56].

The results suggest that medical students’ knowledge on CAM was inadequate. This finding is in line with the results of many studies conducted on medical students in particular and university students in general in many countries including Malaysia [42], Kuwait [43], and Pakistan [57]. The reason for such findings could be the insufficient exposure of medical students to the topic of CAM during their education and training. Therefore, we suggest introducing materials on CAM in the curricula of medical schools.

Additionally, the differences in knowledge between medical students based on their year of study, type of residency, and their families’ monthly income were found to be significant in the results of this study. The difference in knowledge between students of different study years could be due to the lack of proper education and courses on CAM which does not allow students’ level of knowledge on CAM to culminate as they progress through the years of study and training. Whereas the knowledge difference between students coming from different socioeconomic backgrounds (residency type and monthly income) could be the result of the different types of CAM commonly used in the different backgrounds they come from and the sources of knowledge available in these backgrounds. This could be especially true when we consider the high percentage of students who acquire knowledge on CAM mainly through their relatives and friends.

As for the sources medical students used to acquire their knowledge on CAM, social media and the internet were the most commonly cited sources. Acquiring knowledge on CAM from the media seems to be a common phenomenon in many countries. For, example a study from Germany found that media was the second most common source of knowledge on CAM among medical students [58]. However, the unreliable nature of these sources for scientific knowledge, especially on a topic such as CAM, makes this finding worrying. Also, it further necessitates the need for a proper form of education on CAM such as university courses based on evidence-based science and medicine. Fortunately, this can be facilitated by the positive attitude towards CAM among medical students as was found in this study as well as many other similar studies worldwide [12, 59].

Educating health-care professionals about CAM has an important role in ensuring patients’ safety and improving patients care. To that end, educational initiatives have been carried out in some countries and surveys coming from these countries have been showing a rising prevalence of CAM education in medical schools and residency programs [60–66]. It was also reported that the registration in a CAM course can positively impact knowledge and attitude regarding CAM [67].

### Strengths and limitations

This is the first study to assess knowledge, attitude, beliefs, and perception regarding CAM among Palestinian medical students. However, this study suffered from some limitations. These include the use of a convenience sampling technique, which may have led students who are more interested in CAM to step in and participate in the study more frequently than those who are less interested in CAM. Another limitation was the single-center setting of the study. Also, despite the good response rate that we got, our sample size was relatively small. Another limitation of this study was its cross-sectional design, which did not allow assessing the change or the lack thereof in the students’ knowledge and attitude over time. Another potential limitation of the current study was its use of a non-validated questionnaire. This study can be considered an initial approach in evaluating the CAM knowledge, attitudes, and beliefs among Palestinian medical students. Future studies should be performed using validated questionnaires in order to evaluate these parameters.

## Conclusions

In conclusion, the findings of this study suggest that medical students’ knowledge on CAM was inadequate and their perception and attitudes regarding CAM was mixed. Therefore, we suggest integrating material on CAM in the curriculum of medical students in order to bridge their knowledge gap and acquire the necessary tools to meet the patients’ expectations and needs in relation to CAM use. Considering this study’s inability to clarify the causation relationship and the impact of CAM course on students’ attitude, practice, or knowledge, we encourage future prospective studies to explore these grounds.

## Additional file

Additional file 1: Study questionnaire. This is the final version of the English and Arabic version that was used to obtain data which will help to assess Complementary and alternative medicine (CAM) knowledge, attitudes, and beliefs among Palestinian medical students. (DOCX 36 kb)

## Abbreviations

ANNU: An-Najah National University
CAM: Complementary and Alternative Medicine
IRB: Institutional Review Board
TCM: Traditional Chinese Medicine
