# Supplementary material for: Use and acceptance of complementary and alternative medicine among medical students: a cross sectional study from Palestine
Source: BMC Complement Altern Med. 2019 Apr 2;19:78. doi: 10.1186/s12906-019-2492-x (PMC6444598; doi:10.1186/s12906-019-2492-x)
Supplement: Supplementary file 1 — Study questionnaire. This is the final version of the English and Arabic version that was used to obtain data which will help to assess Complementary and alternative medicine (CAM) knowledge, attitudes, and beliefs among Palestinian medical students. (DOCX 36 kb) [file 12906_2019_2492_MOESM1_ESM.docx]

**Additional file 1: Study questionnaires. This is the final version of the English and Arabic version that was used to obtain data which will help to assess Complementary and alternative medicine (CAM) knowledge, attitudes, and beliefs among Palestinian medical students.**

**English version**

**Part one:** **Demographic Characteristics of participants**

1. **Age** __________. 2. Male ____, Female ____. 3. **Year of study**: 4^th^ ____, 5^th^ ____, 6^th^ ____.

4. **Locality you grew up in**: Rural ____, Urban ____, Camp ____.

5. **In what category does your family’s monthly income fall?**

| 1- Less than 500 JD | 2- 500-1000 JD | 3- 1000-2000 JD | 4- More than 2000 JD |
| --- | --- | --- | --- |

**Part two:** **Knowledge regarding Complementary and Alternative Medicine (CAM)**

**1. Please answer the following questions with (yes), (no), or (I don’t know).**

| **No.** | **Question** | **Yes** | **No** | **I don’t know** |
| --- | --- | --- | --- | --- |
| 1 | Senna contraindicated in case of pregnancy and children under 12 years. |  |  |  |
| 2 | Eating Spinach is safe for kidney patients. |  |  |  |
| 3 | Fenugreek increases the risk of elevated blood sugar so it should be avoided in diabetes patients. |  |  |  |
| 4 | Garlic increase the possibility of bleeding when used with warfarin. |  |  |  |
| 5 | Bronchoconstriction is a side effect of caffeine. |  |  |  |
| 6 | Ginger is effective in decreasing PMS. |  |  |  |
| 7 | Echinacea is used to suppress immunity. |  |  |  |
| 8 | The use of digoxin with bran will increase the concentration of digoxin. |  |  |  |

**2. Please complete the following table carefully.**

| **Term** | **1. Is it familiar to you?** | **2. Have you ever used it?** | **3. Would you advice using it to patients?** |
| --- | --- | --- | --- |
| Homeopathy |  |  |  |
| Naturopathy |  |  |  |
| Acupuncture |  |  |  |
| Ayurvedic medicine |  |  |  |
| Aromatherapy |  |  |  |
| Chiropractic |  |  |  |
| Faith healing |  |  |  |
| Massage therapy |  |  |  |
| Traditional African Healing |  |  |  |
| Iridology |  |  |  |
| Hypnosis |  |  |  |
| Meditation |  |  |  |
| Yoga |  |  |  |
| Reflexology |  |  |  |
| Energy Medicine |  |  |  |
| Herbal medicine |  |  |  |
| Biofeedback |  |  |  |

**3. In your own experience, which of the following sources provided you with information on CAM? (You can select more than one source.)**

| **No.** | **Source** | **Select by drawing a mark** |
| --- | --- | --- |
| 1 | Doctor |  |
| 2 | Relatives |  |
| 3 | Pharmacist |  |
| 4 | Social media |  |
| 5 | TV |  |
| 6 | Mosque |  |
| 7 | Friends |  |
| 8 | Internet |  |
| 9 | Scientific magazines |  |
| 10 | Religious books |  |
| 11 | School |  |
| 12 | University |  |
| 13 | Scientific books |  |
| 14 | Others |  |

**Part three:** **Attitude and beliefs towards Complementary and Alternative Medicine (CAM)**

**1. Please draw a mark under the appropriate answer for each of the following questions regarding attitude towards Complementary and Alternative Medicine (CAM).**

| **No.** | **Question** | **Yes** | **No** | **No opinion** |
| --- | --- | --- | --- | --- |
| 1 | Do you believe that all types of CAM are beneficial to healthcare? |  |  |  |
| 2 | As a future doctor, will you recommend CAM to a patient? |  |  |  |
| 3 | Do you agree that patients have right to choose between CAM and orthodox medicine? |  |  |  |
| 4 | As a future doctor, will you encourage the use of CAM together with OM? |  |  |  |
| 5 | Do you agree that CAM should be introduced in medical course? |  |  |  |
| 6 | Will you be ready to be trained more on CAM after becoming a doctor? |  |  |  |
| 7 | Do you agree that it's necessary to ask every patient of previous usage of CAM during history taking? |  |  |  |
| 8 | As a future doctor, will you ask patient of previous CAM use? |  |  |  |
| 9 | Do you agree that It is necessary for a doctor to have good knowledge of CAM? |  |  |  |
| 10 | As a future doctor, will you have a positive reaction should a patient ask you to recommend a CAM? |  |  |  |

**2. Please draw a mark under the appropriate answer for each of the following items based on whether you, as a future doctor, will likely recommend each items to a patient or not.**

| **No.** | **Will you recommend this item to a patient?** | **Yes** | **No** | **No opinion** |
| --- | --- | --- | --- | --- |
| 1 | Exercises |  |  |  |
| 2 | Supplements |  |  |  |
| 3 | Honey |  |  |  |
| 4 | Massage |  |  |  |
| 5 | Herbs |  |  |  |
| 6 | Quran |  |  |  |
| 7 | Fasting |  |  |  |
| 8 | Praying |  |  |  |
| 9 | Hejama |  |  |  |
| 10 | Zamzam |  |  |  |
| 11 | Remedies |  |  |  |
| 12 | Music |  |  |  |
| 13 | Chiropractic |  |  |  |
| 14 | Acupuncture |  |  |  |
| 15 | Aromatherapy |  |  |  |
| 16 | Bloodletting |  |  |  |
| 17 | Cauterization |  |  |  |
| 18 | Animal products |  |  |  |
| 19 | Reflexology |  |  |  |

**3. If you are to recommend a CAM modality/ item, which of the following would you consider as a motivating factor to your decision? (You can choose more than one factor.)**

| **No.** | **Factor** | **Yes** | **No** |
| --- | --- | --- | --- |
| 1 | Product efficacy is scientifically proven |  |  |
| 2 | Positive responses from patients on the effectiveness of the product |  |  |
| 3 | Fewer side effects |  |  |
| 4 | Less expensive (Cheaper) |  |  |
| 5 | Publicity of the product |  |  |
| 6 | Highest profit |  |  |
| 7 | Incentives from manufacturers |  |  |

**4. Please draw a mark under the appropriate answer as to which degree you agree/ disagree with each of the following statements.**

| **No.** | **Statement** | **Strongly agree** | **Agree** | **Disagree** | **Strongly disagree** | **No opinion** |
| --- | --- | --- | --- | --- | --- | --- |
| 1 | All kinds of complementary medicine are safe and have very few side effects |  |  |  |  |  |
| 2 | Conventional medicine doesn't offer the patient benefits offered by alternative medicine |  |  |  |  |  |
| 3 | Results of complementary medicine is mainly due to placebo effect |  |  |  |  |  |
| 4 | I have full trust to debate with patients about terms of alternative and complementary medicine |  |  |  |  |  |
| 5 | Complementary and alternative medicine not only cure the disease but will improve general health in other hand |  |  |  |  |  |
| 6 | The doctor should continuously question whether the patient was used modalities of alternative medicine |  |  |  |  |  |
| 7 | We need scientific evaluation before use complementary and alternative medicine |  |  |  |  |  |

**5. Please answer with (yes) or (no) as to which of the following you believe to be a barrier that limit the appropriate use of complementary and alternative medicine (CAM).**

| **No.** | **Do you believe this to be a barrier to the use of CAM?** | **Yes** | **No** | **No opinion** |
| --- | --- | --- | --- | --- |
| 1 | Small number of trained personnel to use CAM |  |  |  |
| 2 | Lack of scientific knowledge in CAM |  |  |  |
| 3 | Lack of scientific evidence to use CAM |  |  |  |
| 4 | Lack of reliable sources of information |  |  |  |
| 5 | Need a long time of treatment |  |  |  |
| 6 | Lack of time |  |  |  |
| 7 | Lack of interest in CAM |  |  |  |
| 8 | There is no obstacle |  |  |  |

**Arabic version**

**هذا الاستبيان يقوم به طلبة الطب البشري في جامعة النجاح الوطنية لغرض البحث العلمي, لتقييم المعرفة والسلوك والاستخدام فيما يتعلق بموضوع الطب المكمّل والبديل ضمن طلبة الطب في فلسطين. نرجو الإجابة عن جميع الأسئلة بدقة, علماً بأن هذه المعلومات ستستخدم لأغراض البحث العلمي فقط.**

**الجزء الأول : الخصائص السكانية (الديموغرافية) للمجيبين :**

**العمر** ____

**الجنس:**

1-ذكر 2-أنثى

**السنة الدراسية :**

1- الرابعة 2-الخامسة 3-السادسة

**في أي مكان نشأت ؟**

1-المدينة 2-الريف 3-المخيم

**معدل دخل العائله الشهري؟**

1-اقل من 500 دينار 2- من 500-1000 3- من 1000-2000 4- اكثر من 2000

**الجزء الثاني: مدى معرفتك حول الطب البديل والطب المكمل:**

1.**هل مصطلح الطب البديل والطب المكمل مألوف لك ؟**

1-نعم 2-لا

2**.أرجو أن تجيب عن الأسئلة التالية ب(نعم)، (لا) أو (لا أعلم) من خلال وضع اشارة صح في المكان المخصص**:

1**. من فصلك، أجب على الأسئلة التالية بـ (نعم)، (لا)، أو (لا أعلم).**

| **لا أعلم** | **لا** | **نعم** | **السؤال** | **الرقم** |
| --- | --- | --- | --- | --- |
|  |  |  | يمنع استخدام (السنا) من قبل الحوامل و الأطفال تحت سن ال12 ؟ | 1 |
|  |  |  | أكل السبانخ آمن لمرضى السكري؟ | 2 |
|  |  |  | ترفع نبتة الحلبة مستوى السكر في الدم وبالتالي يجب منع مرضى السكر من تناولها | 3 |
|  |  |  | الثوم يزيد من احتمالية النزيف عند استخدامه مع دواء مميع الدم (Warfarin) | 4 |
|  |  |  | تضيق القصبات الهوائية هو عارض جانبي لاستخدام الكافيين | 5 |
|  |  |  | يفيد الزنجبيل في تخفيف متلازمة ما قبل الحيض | 6 |
|  |  |  | تستخدم زهرة الاشنسا في تثبيط جهاز المناعة | 7 |
|  |  |  | استخدام دواء (Digoxin) مع النخالة يزيد من تركيز الدواء في الدم | 8 |

2**. من فضلك، أكمل الجدول التالي بدقة. (للسؤال الثالث، يمكن الاختيار من بين التالية: التلفاز، الأقارب والأصدقاء، المدرسة، الكتب والمجلات العلمية، وسائط الإعلام، المسجد أو الكنيسة، الأنترنت، المسؤسسات الصحة، إلخ.)**

| **4. إين سمعت عنه لأول مرة؟** | **3. هل تنصح به للمرضى** | **2. هل استخدمته من قبل؟** | **1. هل هو مألوف لك؟** | **المصطلح** |
| --- | --- | --- | --- | --- |
|  |  |  |  | المعالجة المثلية |
|  |  |  |  | مداواة طبيعية |
|  |  |  |  | العلاج بالإبر |
|  |  |  |  | طب الايورفيردا |
|  |  |  |  | طب العطارين |
|  |  |  |  | تقويم العمود الفقري الطبيعي |
|  |  |  |  | العلاج بالايمان |
|  |  |  |  | العلاج بالمساج |
|  |  |  |  | العلاج الافريقي التقليدي |
|  |  |  |  | علم القزحية |
|  |  |  |  | التنويم المغناطيسي |
|  |  |  |  | التأمل |
|  |  |  |  | اليوغا |
|  |  |  |  | العلاج عن طريق نقاط الارتكاز |
|  |  |  |  | طب الطاقة |
|  |  |  |  | طب الاعشاب |
|  |  |  |  | الارتجاع البيولوجي |

**3. من خلال تجربتك الشخصية، أي المصادر التالية زودتك بمعلومات عن الطب المكمل والبديل؟ (يمكنك اختيار أكثر من مصدر واحد.)**

| **اختر بوضع اشارة** | **المصدر** | **الرقم** |
| --- | --- | --- |
|  | الطبيب | 1 |
|  | الأقارب | 2 |
|  | الصيدلاني | 3 |
|  | وسائط التواصل الاجتماعي | 4 |
|  | التلفاز | 5 |
|  | المسجد | 6 |
|  | الأصدقاء | 7 |
|  | الإنترنت | 8 |
|  | المجلات العلمية | 9 |
|  | الكتب الدينية | 10 |
|  | المدرسة | 11 |
|  | الجامعة | 12 |
|  | الكتب العلمية | 13 |
|  | غير ذلك | 14 |

**القسم الثالث: الموقف من الطب المكمل والبديل والمعتقدات المتعلقة به**

**1. من فضلك، ضع إشارة تحت الإجابة المناسبة لكل من الأسئلة التالية المتعلقة بالموقف من الطب المكمل والبديل.**

| **لا رأي** | **لا** | **نعم** | **السؤال** | **الرقم** |
| --- | --- | --- | --- | --- |
|  |  |  | هل تعتقد أن جميع أنواع الطب المكمل والبديل مفيد للرعاية الصحية؟ | 1 |
|  |  |  | كطبيب في المستقبل، سوف توصي بالطب التكميلي والبديل للمريض؟ | 2 |
|  |  |  | هل توافق على أن المرضى لديهم الحق في الاختيار بين الطب التكميلي والبديل اوالطب النبوي ؟ | 3 |
|  |  |  | كطبيب في المستقبل، سوف تشجع استخدام الطب التكميلي والبديل جنبا إلى جنب مع الطب النبوي ؟ | 4 |
|  |  |  | هل توافق على أنه ينبغي تعليم الطب البديل في الكليات الطبية؟ | 5 |
|  |  |  | هل ستكون على استعداد للتدرب أكثر على الطب البديل بعد أن تصبح طبيبا؟ | 6 |
|  |  |  | هل توافق على أنه من الضروري أن تسأل كل مريض عن الاستخدام السابق للطب البديل خلال أخذ التاريخ المرضي ؟ | 7 |
|  |  |  | كطبيب في المستقبل، سوف تسأل المريض من استخدام الطب البديل في السابق؟ | 8 |
|  |  |  | هل توافق على أنه من الضروري أن يكون لدى الطبيب معرفة جيدة بالطب التكميلي والبديل ؟ | 9 |
|  |  |  | كطبيب في المستقبل، سيكون لديك رد فعل إيجابي لمريض يطلب منك أن تعطيه تعليمات عن الطب التكميلي والبديل ؟ | 10 |

2**. من فضلك، ضع إشارة تحت الإجابة المناسبة لكل من البنود التالية بناء على فيما إذا كنت من المحتمل أن توصي بكل بند من البنود للمرضى أم لا كطبيب مستقبلي.**

| **لا رأي** | **لا** | **نعم** | **هل ستوصي بهذا البند لمريض؟** | **الرقم** |
| --- | --- | --- | --- | --- |
|  |  |  | التمارين الرياضية | 1 |
|  |  |  | المكملات الغذائية | 2 |
|  |  |  | العسل | 3 |
|  |  |  | المساج | 4 |
|  |  |  | الأعشاب | 5 |
|  |  |  | القرآن | 6 |
|  |  |  | الصيام | 7 |
|  |  |  | الصلاة | 8 |
|  |  |  | الحجامة | 9 |
|  |  |  | ماء زمزم | 10 |
|  |  |  | الوصفات العلاجية الطبيعية | 11 |
|  |  |  | الموسيقى | 12 |
|  |  |  | تقويم العمود الفقري الطبيعي | 13 |
|  |  |  | العلاج بالإبر | 14 |
|  |  |  | طب العطارين | 15 |
|  |  |  | سحب الدم | 16 |
|  |  |  | العلاج بالكي | 17 |
|  |  |  | المنتجات الحوانية | 18 |
|  |  |  | العلاج عن طريق نقاط الارتكاز | 19 |

3**. إذا كنت لتوصي باستخدام أحد أنواع الطب المكمل والبديل، أي التالية تعتبره عامل محفز لك عن اتخاذ هذا القرار؟ (تستطيع اختيار أكثر من خيار واحد.)**

| لا | نعم | العامل | الرقم |
| --- | --- | --- | --- |
|  |  | فعالية المنتج المثبتة علميا | 1 |
|  |  | استجابة المرضى الإيجابية لفعالية المنتج | 2 |
|  |  | أعراض جانبية قليلة | 3 |
|  |  | أقل تكلفة | 4 |
|  |  | شعبية المنتج | 5 |
|  |  | نسبة الربح العالية | 6 |
|  |  | المحفزات من قبل المصنعين | 7 |

**4. من فضلك، ضع إشارة تحت الإجابة المعبرة عن درجة اتفاقك مع كل من الجمل التالية.**

| لا رأي | لا أتفق بشدة | لا أتفق | أتفق | أتفق بشدة | الجملة | الرقم |
| --- | --- | --- | --- | --- | --- | --- |
|  |  |  |  |  | كل أنواع الطب المكمل آمنة ولها آثار جانبية قليلة جدا | 1 |
|  |  |  |  |  | الطب البديل يقدم فوائد للمريض لا يقدمها الطب التقليدي | 2 |
|  |  |  |  |  | نتائج الطب التكميلي هي أساسا بسبب تأثير الدواء الوهمي | 3 |
|  |  |  |  |  | لدي الثقة الكاملة لمناقشة شروط الطب البديل والتكميلي مع المرضى | 4 |
|  |  |  |  |  | سيؤدي الطب التكميلي والبديل إلى تحسين الصحة العامة وليس فقط علاج المرض | 5 |
|  |  |  |  |  | يجب أن يتساءل الطبيب باستمرار عما إذا كان المريض قد استخدم أنواع الطب البديل | 6 |
|  |  |  |  |  | يحتاج الطب التكميلي والبديل التقييم العلمي قبل الاستخدام | 7 |

**5. من فضلك، أجب بـ (نعم) أو (لا) بما يتناسب مع فيما إذا كنت تعتقد إن التالية تشكل عائقة يحد من الاستخدام المناسب للطب المكمل والبديل.**

| لا رأي | لا | نعم | هل تعتقد إن هذا عائق لاستخدام الطب المكمل والبديل؟ | الرقم |
| --- | --- | --- | --- | --- |
|  |  |  | قلة عدد الأشخاص المدربين على استخدام الطب المكمل والبديل | 1 |
|  |  |  | نقص المعرفة العلمية المتعلقة بالطب المكمل والبديل | 2 |
|  |  |  | نقص الشواهد العلمية على استخدام الطب المكمل والبديل | 3 |
|  |  |  | نقص مصادر المعلومات الموثوقة | 4 |
|  |  |  | الحاجة لمدة علاج طويلة | 5 |
|  |  |  | قلة الوقت | 6 |
|  |  |  | قلة الاهتمام بالطب المكمل والبديل | 7 |
|  |  |  | لا يوجد عوائق | 8 |
